# Design of and Early Insights From a Generalized Myasthenia Gravis Patient Engagement Research Council

**DOI:** 10.1002/hsr2.70230

**Published:** 2024-12-17

**Authors:** Gabrielle Geonnotti, Jacqueline Pesa, Wesley Peters, Melina Taylor, Zia Choudhry, Oluyemisi Falope, Marquetta Price, Lucy Baxter, Bruce West, Lisa Shea

**Affiliations:** ^1^ Janssen Scientific Affairs, LLC Titusville New Jersey USA; ^2^ Evidera Wilmington North Carolina USA; ^3^ Generalized Myasthenia Gravis Patient Engagement Research Council participant USA

**Keywords:** caregivers, myasthenia gravis, patient engagement, patient‐reported outcomes

## Abstract

**Background and Aims:**

An exploratory focus group study was conducted to better understand the needs of patients living with generalized myasthenia gravis (gMG).

**Methods:**

US‐based adults with gMG and caregivers of patients with gMG participated in a Patient Engagement Research Council between August 2022 and January 2023. The study consisted of a 15‐min prework survey, followed by virtual focus groups facilitated using a semi‐structured interview guide. Data concepts were identified using conversational analysis and by direct observation. All transcripts were coded based on concepts using a qualitative research analysis program (MaxQDA).

**Results:**

16 participants (13 patients, three caregivers) were recruited. Participants reported impact on daily activities, fatigue, and psychosocial problems. Many participants experienced delayed diagnosis and difficulty accessing specialist care. Participants described multiple barriers related to their gMG, including barriers to treatment, access‐related issues, and communication disconnect between patients and healthcare professionals. Achieving stable disease was the most important goal. There was a preference for the autonomy of self‐administered medications at home versus infusions. Study insights led to recommendations to guide patient and healthcare professional education.

**Conclusion:**

The study illustrates the need to improve access to specialist care, achieve earlier diagnosis, prioritize patients' preferences in disease management, and develop treatments that improve outcomes without additional burden.

**Patient or Public Contribution:**

The data collected in this study was provided by the focus group participants, which included patients and caregivers of those with myasthenia gravis.

## 1 Introduction

Myasthenia gravis (MG) is a rare, incurable, autoantibody‐mediated disorder characterized by fatigue and muscle weakness [1]. Ocular weakness is the most common initial presentation, but progression to generalized MG (gMG) involving other muscle systems occurs within approximately 3 years in most patients [2]. Many patients report gMG has a significant impact on their daily living and overall quality of life [3, 4], and it is associated with substantial caregiver burden [5]. Patients with gMG experienced worse health‐related quality of life compared to the general population [6], and patients report struggling with everyday life due to either their disease severity or perspective concerning gMG [7]. In women, the incidence of MG is highest between the ages of 30 and 50 years; in men, incidence increases with age [8]. The clinical characteristics and treatment of MG are similar in older and younger patients, but there is an increased risk of medication adverse effects and comorbidities in older patients (with onset after 50 years of age) [9]. Some racial differences in MG have been reported, with an earlier onset and tendency towards more severe disease in Black/African American compared with White patients [10].

Studies have shown that up to 85% of patients with gMG have autoantibodies directed against the acetylcholine receptor, while autoantibodies directed against muscle‐specific kinase are found in approximately 6% of patients, and patients without autoantibodies are considered to be seronegative [1, 11, 12]. Autoantibodies against several other targets have been detected in patients previously thought to be seronegative [12].

Acetylcholinesterase inhibitors, along with immunosuppressive corticosteroids and immunomodulatory agents, are the mainstay of first‐line MG treatments [11, 13]. In addition, intravenous immunoglobulin (IVIg) and therapeutic plasma exchange are used to achieve rapid relief during flare‐ups [11]. Surgery to remove the thymus has been shown to benefit certain patients and those with a thymoma [2]. However, these nonspecific therapies are often associated with side effects that impact patients' quality of life. As knowledge about the disease increases, there is an effort to replace and supplement these traditional therapies with more effective and tolerable treatments, with the aim of improving patient outcomes and quality of life [12]. Seronegative patients in particular have limited treatment options [14]. While some seronegative patients have been given special approval from their insurance companies to receive newer treatments off‐label, these options are commonly only available for seropositive patients with gMG (including new options for both muscle‐specific tyrosine kinase positive and acetylcholine receptor positive serotypes). Although the treatment landscape for gMG is expected to shift in the coming years due to diagnostic advances, increased disease awareness, and the introduction of emerging therapies, currently ~30%–40% of patients experience no meaningful improvement from existing treatment options [15].

With good management and optimized symptomatic and immunosuppressive treatment, the long‐term prognosis for gMG patients is generally good, but there remains a need to improve overall quality of life and achieve disease stability [3]. Approximately 10% of patients do not respond adequately to, or are intolerant of, treatment, and only 20% attain stable remission [16]. Disease instability has a major effect on patient quality of life [17, 18]. Furthermore, approximately 15–20% of patients with MG experience a myasthenic crisis during the course of their disease, with associated severe weakness of respiratory and bulbar muscles that requires hospitalization and intubation or respiratory support [19]. Mortality associated with a myasthenic crisis has been reported in 4–12% of cases, and some survivors continue to experience substantial burden [20, 21, 22, 23].

Only a few studies have looked at the experiences of patients living with gMG and how these experiences can be drawn upon to improve patient care [17, 18, 24]. For example, a recent study in Japan indicated 27% of Japanese patients with MG are dissatisfied with their life and highlights the need for more effective treatments targeting broad MG serotypes [25]; however, there is a paucity of similar studies in the United States. Patients and caregivers (usually a family member or friend with patient care responsibilities) possess experiential knowledge and lived experience that is unique and complementary to clinical development and real‐world research. Their perspectives can improve research quality and relevance, as well as patient outcomes [26, 27]. Through an exploratory focus group study with a diverse range of participants, our goal was to better understand the patient experience from first symptoms to diagnosis and subsequent treatment to help identify unmet needs of patients with gMG. This article describes the results from the initial focus group and provides recommendations for the care of patients with gMG.

## 2 Methods

To gather the perspectives of people living with and affected by gMG, patients and caregivers were recruited into a gMG Patient Engagement Research Council (PERC) established by Janssen. PERCs consist of diverse groups of disease‐aware patients and caregivers with certain health conditions whose knowledge can inform research [28, 29, 30, 31].

### 2.1 Selection of gMG PERC Participants

Eligible participants were ≥ 18 years old and resided in the United States. They were a patient with a self‐reported diagnosis of gMG or a caregiver to a patient meeting the eligibility criteria; pairs of a patient and their caregiver were not considered. Participants were recruited to the PERC between August 2022 and January 2023.

Potential participants were screened for inclusion using a gMG‐specific online screener, followed by a telephone interview. Those eligible were then selected to ensure diversity in age, gender, and race/ethnicity, as well as to obtain variety in time since gMG diagnosis, disease severity, treatment experiences, and age at diagnosis (early adulthood [age 20–30 years] or later in life [age 50–60 years]).

### 2.2 Procedures

**Figure 1:**
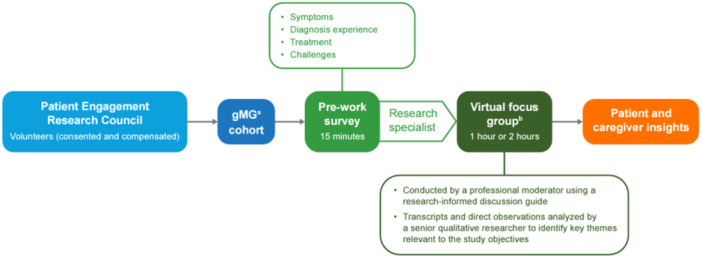
Summary of the study design, relevant patients included, and analysis of the Patient Engagement Research Council. ^a^Self‐reported diagnosis. ^b^Also attended by at least one relevant member of Janssen's medical/scientific team. gMG, generalized myasthenia gravis.

Participants were informed that participation was voluntary, responses would be recorded, no treatments would be provided, and they could withdraw at any time. They signed a consent and release form that communicated confidentiality and Health Insurance Portability and Accountability Act–compliant practices. The study consisted of a 15‐min prework survey, followed by virtual focus groups (Figure 1). The prework survey used open‐ended questions to gather information on initial and current symptoms, diagnosis, and medications taken for gMG. All participants engaged in a 1‐h introductory virtual session; patients and caregivers then participated in one virtual focus group (two separate 2‐h sessions were held with patients and a 1‐h session was held with caregivers). The patient sessions were longer due to a greater number of patients than caregivers in the PERC. The prework survey, materials presented to facilitate discussion, and discussion prompts were identical for each group. Each session was attended by at least one member of Janssen's medical/scientific team. Group sessions were conducted by a professional moderator using a research‐informed discussion guide. Sessions were audio‐recorded and transcribed.

The purpose of the focus group was to introduce participants to the PERC program and to explore patient perceptions around several concepts in the context of their disease journey: first and ongoing symptoms (fluctuations and most bothersome), experience with receiving a diagnosis/potential misdiagnosis, activities of daily living, quality of life, impact on work/school, and outcomes of importance, along with their overall treatment experience including the perceived burden of treatments.

### 2.3 Data Analysis

Concepts in the data were identified by a senior qualitative researcher, with support from a research associate, using conversational (narrative) analysis and by direct observation, both during data collection and through transcript analysis. Data analysis consisted of applying a thematic analysis framework to review and code transcripts based on a priori topics and lived experiences of participants using a qualitative research analysis program (MaxQDA). A set of recommendations, based on key insights from, and discussions with, the patients and caregivers during the focus group, was developed in an initial debrief meeting to review high‐level findings and recommendations; the recommendations were consolidated in the formal final report presenting the findings from the PERC discussions. Demographic data were collated and descriptively analyzed.

## 3 Results

### 3.1 PERC Participants and Demographics

Key demographics of the participants are summarized in Table 1. In total, there were 13 patients and three caregivers with a mean age of 54.1 years. There were 10 females (62.5%), five males (31.3%), and one participant (in the patient group) who identified as nonbinary (6.3%). Racial/ethnic identities of the participants were White (*n* = 10 [62.5%]), Black/African American (*n* = 4 [25.0%]), American Indian/Alaska Native (*n* = 1 [6.3%]), and Hispanic/Latino (*n* = 1 [6.3%]). Two patients self‐reported seronegativity for anti‐acetylcholine receptor antibodies and/or anti‐muscle‐specific receptor tyrosine kinase antibodies. The majority of participants had a high level of education—three had a graduate degree (18.8%) and seven had a bachelor's degree (43.8%), while the rest had some form of college education, went to a technical or trade school, or had a high school diploma. Most of the participants lived in a suburban area (68.8%) or a rural area (25.0%), with one from an urban area (6.3%). These corresponded to various locations across the United States: Northeast (6.3%), Southeast (62.5%), Midwest (6.3%), West (6.3%), and Southwest (18.8%). The time since gMG diagnosis also varied from 1 to 5 years (*n* = 8 [50.0%]), 6–10 years (*n* = 4 [25.0%]), and ≥ 21 years (*n* = 4 [25.0%]).

**Table 1:** Key demographics and characteristics of gMG PERC participants.

Characteristic, *n* (%)	Number (%) of participants (*N* = 16)
Gender	
Female	10 (62.5)
Male	5 (31.3)
Nonbinary	1 (6.3)
Race/ethnicity	
White	10 (62.5)
Black/African American	4 (25.0)
American Indian/Alaska Native	1 (6.3)
Hispanic/Latino	1 (6.3)
Age group, years	
18–34	1 (6.3)
35–54	8 (50.0)
55–64	3 (18.8)
65–69	4 (25.0)
Education	
Graduate degree	3 (18.8)
Bachelor's degree	7 (43.8)
Some college	2 (12.5)
Technical or trade school	1 (6.3)
High school diploma	3 (18.8)
Locality^a^	
Urban	1 (6.3)
Suburban	11 (68.8)
Rural	4 (25.0)
US region	
Southeast	10 (62.5)
Southwest	3 (18.8)
West	1 (6.3)
Midwest	1 (6.3)
Northeast	1 (6.3)
Northwest	0
Time since diagnosis, years^b^	
1–5	8 (50.0)
6–10	4 (25.0)
11–15	0
16–20	0
≥ 21	4 (25.0)

Abbreviations: gMG, generalized myasthenia gravis; PERC, Patient Engagement Research Council.

^a^ Urban areas consist of both living and working areas with a high population or are zip codes in large cities; suburban areas are mainly residential with a larger population than rural areas; rural areas are open and spread out with a small population.

^b^ For caregivers, the time shown represents the time since diagnosis of the patient they care for.

### 3.2 Findings from the Prework Survey

**Figure 2:**
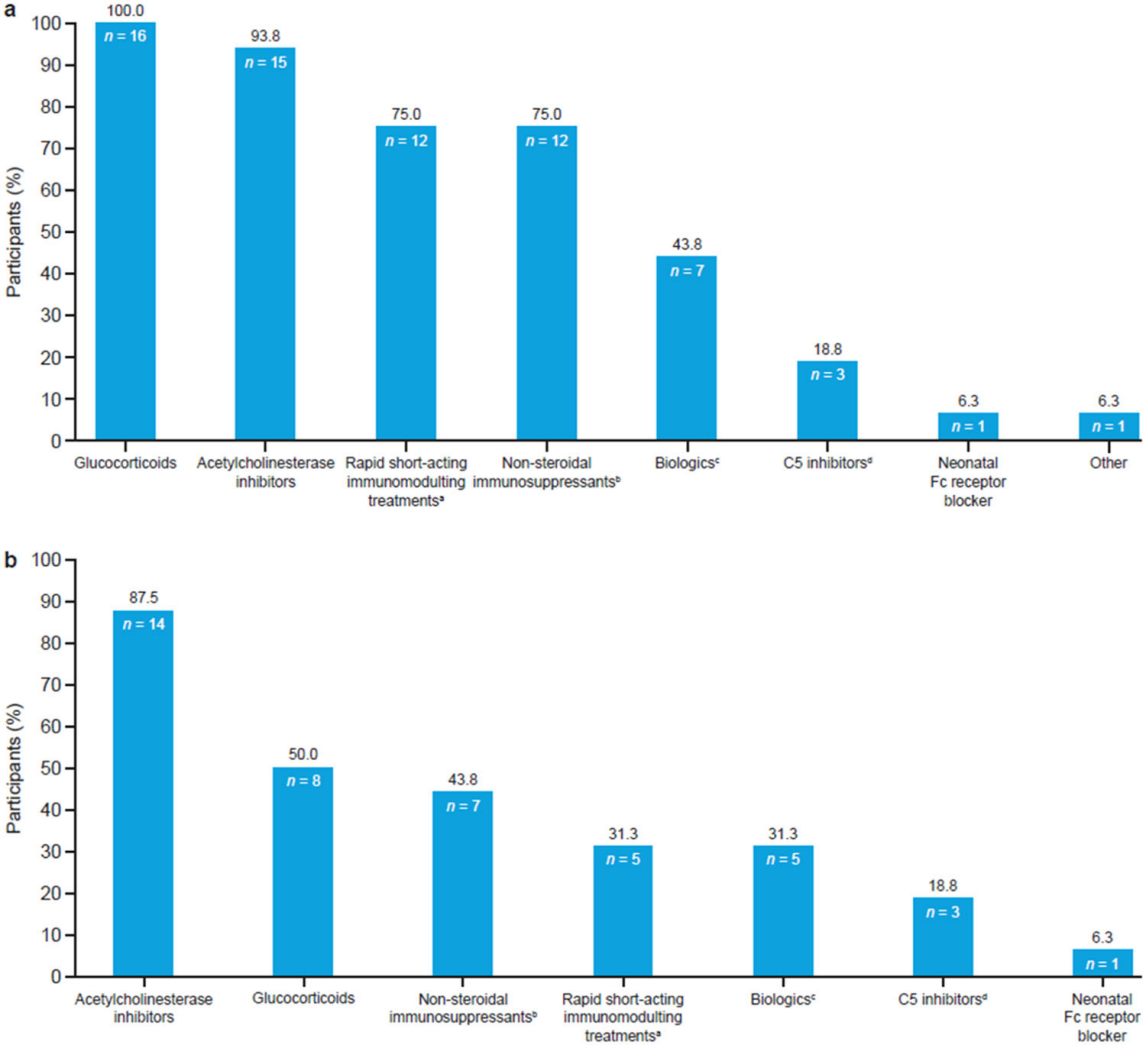
Treatment regimens for gMG among PERC participants. (a) Past treatment regimens; (b) current treatment (i.e., at the time of the survey). ^a^For example, plasma exchange or intravenous immunoglobulin. ^b^For example, azathioprine or cyclosporine. ^c^For example, rituximab. ^d^For example, eculizumab. gMG, generalized myasthenia gravis; PERC, Patient Engagement Research Council.

As part of the prework survey, participants were asked about classes of medications they had taken over the course of their gMG (Figure 2a) and were asked about their current treatment (Figure 2b).

The most common prior treatments were glucocorticoids and acetylcholinesterase inhibitors; all participants (*N* = 16 [100.0%]) had experience with glucocorticoid therapy and most (*n* = 15 [93.8%]) had taken acetylcholinesterase inhibitors. Past treatments also included rapid short‐acting immunomodulating treatments (*n* = 12 [75.0%]), nonsteroidal immunosuppressants (*n* = 12 [75.0%]), biologics (*n* = 7 [43.8%]), complement inhibitors (*n* = 3 [18.8%]), neonatal Fc receptor blocker (*n* = 1 [6.3%]), and “other” treatments (*n* = 1 [6.3%]). For current treatment regimens, acetylcholinesterase inhibitors were the most common (*n* = 14 [87.5%]), followed by glucocorticoids (*n* = 8 [50.0%]), nonsteroidal immunosuppressants (*n* = 7 [43.8%]), rapid short‐acting immunomodulating treatments (*n* = 5 [31.3%]), biologics (*n* = 5 [31.3%]), complement inhibitors (*n* = 3 [18.8%]), and neonatal Fc receptor blocker (*n* = 1 [6.3%]).

**Figure 3:**
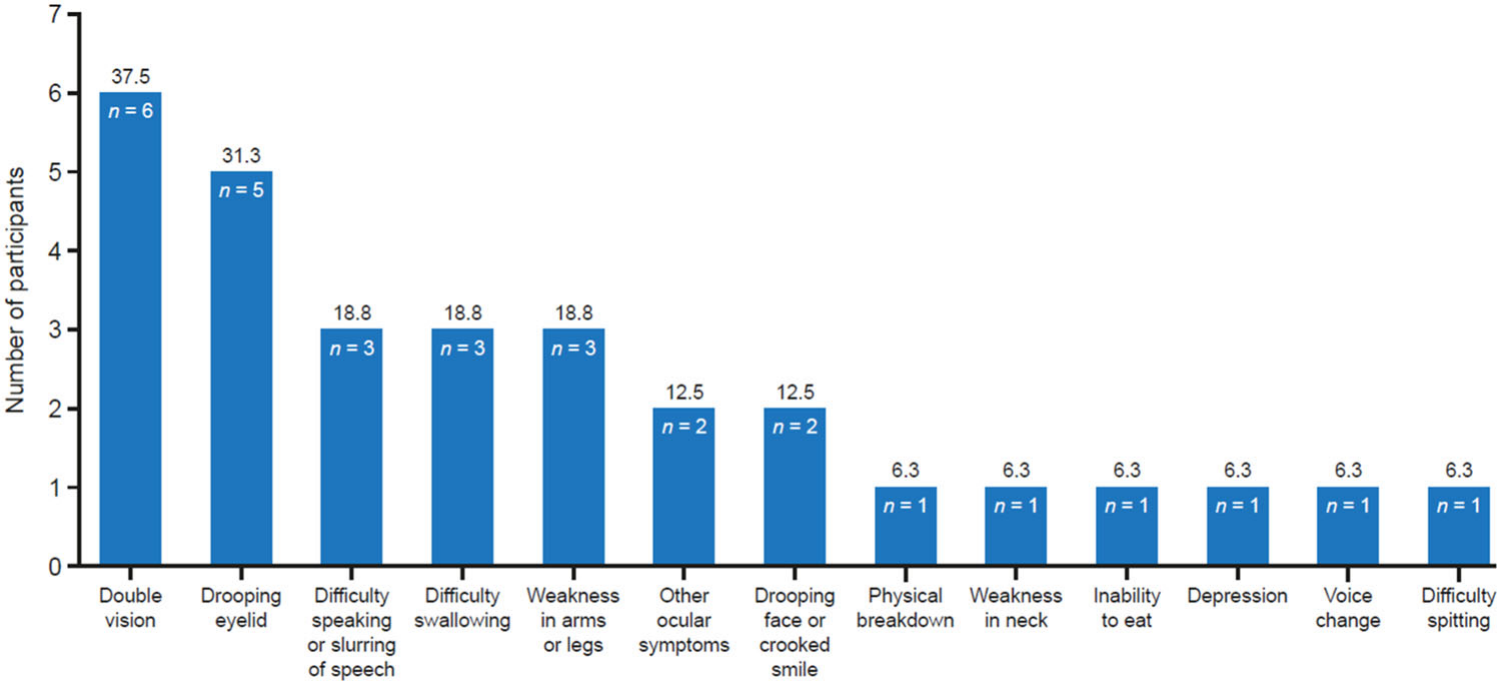
Initial symptoms observed in patients with gMG. The bar chart summarizes the results of the prework survey, highlighting the main initial symptoms of gMG identified by patients themselves. Note that participants were allowed to give more than one answer (there were 30 entries from 16 participants). gMG, generalized myasthenia gravis.

As is common in MG, ocular problems were the most frequent initial symptoms among the gMG PERC participants. Six (37.5%) had experienced double vision, five (31.3%) had a drooping eyelid, and two (12.5%) had other ocular symptoms. Three (18.8%) participants experienced difficulty speaking or slurring of speech, difficulty swallowing, and weakness in arms or legs. Other symptoms occurred in one or two participants (Figure 3).

### 3.3 Findings from the Focus Groups

The discussions from the focus groups are summarized according to key themes. A detailed narrative from one patient describing their journey with gMG is also provided (see Supporting Information). Table 2 covers the diverse range of experiences reported by participants, which impacted their mental health, work life, and family and social aspects.

**Table 2:** Experiences from gMG PERC participants.

Theme	PERC participant experiences shared during focus groups
First gMG symptoms	1. “*She started with a droopy eye and quadruple vision, and then within 3–4 weeks, she was having general MG symptoms such as muscle weakness. She couldn't hold a purse on her shoulder, she was having trouble holding a water bottle*.” [female caregiver, 50–59 years old]
	2. *“I was so tired just from doing basic things that I literally just shut down completely…”* [female patient, 50–59 years old]
	3. *“I wasn't able to swallow food. Food was getting stuck, and I was scared. I didn't want to tell my parents. So I just stopped eating basically. I was losing a lot of weight and my speech was very slurry. My parents thought I was on drugs and I was becoming anorexic when I wasn't eating.”* [female patient, 44–59 years old]
	4. *“Your eye is like half‐closed. You're droopy on the right side of your face…I'm going to walk you down to the ER and get you in right away for a neurological emergency because it looks like you're having a stroke.”* [male patient, 50–59 years old]
Reaching a diagnosis	5. “*We live in a very rural community. Our town's got about 1300 people. So, the closest neurologist, of course, took like 4 months to even get an appointment to see her. And so then in the meantime, like it started getting worse.”* [female patient, 40–49 years old]
	6. “*I went to my [HCP]. She was a newer nurse practitioner in our area, and I explained what was going on. And she was like, this sounds like [multiple sclerosis] (MS). Like, everything you're talk[ing about] – it sounds like MS. We're going to scan you; we're going to figure out what's going on*.” [female patient, 30–39 years old]
	7. “*There's only one neuromuscular specialist I could see. It became very apparent, very quickly, that she did not believe me, because the bloodwork was not abnormal*.” [nonbinary patient, 30–39 years old]
	8. “*I realized it's so easy to be misdiagnosed, especially in the hospital, because in the emergency room, it's not something they look for. They were treating me for a stroke, COPD, hypertension. But now looking back, I see that the signs were all there*.” [male patient, 60–69 years old]
Treatment experience	9. *“My pulmonologist said: ‘most people after they've had it a year or two of diagnosis, they are well controlled, but you need a trilogy ventilator.’ I have to advocate so hard… and I get the bare minimum treatment.”* [female patient, 30–39 years old]
	10. *“We're really looking for something different now because the IVIg makes her feel really horrible and they have to do it very, very slowly on her, because she's really sensitive so it's 6 h a day for 2 days.”* [female caregiver, 50–59 years old]
	11. *“I've just been in and out, in and out of the hospital. Just in December, I was on a ventilator. I'm just having the hardest time. So all of these treatments, the plasma, the IVIg… they help me get out of the hospital, but they're not… I'm not back at my base.”* [female patient, 40–49 years old]
	12. *“But anyway I wish that all the medicine that I take helped more. Like I said, I still experience some symptoms.”* [female patient, 60–69 years old]
	13. *“[The effect of a neonatal Fc receptor blocker] was overnight, I can say. And I purposely was testing myself to see when I got tired. …I felt like I was superman with all this energy.”* [male patient, 60–69 years old]
	14. “*Going to IVIg monthly and once a year taking the biologic, having to go to the infusion center is inconvenient but it's worth it. It would be nice if it could be either done at home or not as often*.” [male patient, 60–69 years old]
	15. “*When [my neurologist] saw me, he says to me, ‘I can now treat you because you have different insurance.’ I was so upset because I went three additional years untreated because insurance wouldn't allow him to even try. That day he gave me the pills to try out for gMG. The next day, I felt almost like myself*.” [female patient, 50–59 years old]
	16. “*As someone that is seronegative, there's a lot of treatment barriers. I'm getting the minimum treatment options because my insurance won't pay for a lot of the other medications because they're directed toward the acetylcholine receptor positive group. And without me having the antibodies, I can't receive some of the state‐of‐the‐art treatment*.” [female patient, 30–39 years old]
	17. “*It is sometimes hard to help them [doctors and nurses] to understand what's going on with the patient. But that's why I felt like being at the hospital with her all the time was important so that I could be her voice when she couldn't speak*.” [female caregiver, 60–69 years old]
Impact and unmet needs	18. “*Well, the most important goal is to control the symptoms, and obviously I want to go into a remission – completely get rid of myasthenia. But I'd have them keep it under control so I can just live a normal, happy life and do everything with my family, do the things that they do*.” [male patient, 40–49 years old]
	19. “*If I could get half of me back, I would be OK with that… I would absolutely be OK with half of the person that I used to be. I'm not greedy*.” [female patient, 50–59 years old]
	20. “*I'm so sick of so many medications. If only you could go to your fridge and just get something and give it to yourself. Because going to the hospitals, it's so draining*…” [female patient, 40–49 years old]
	21. “*During the summer months, I have to be out [cutting grass] at 7:30am. And my wife has to come out and drag me in the house. If I were to stay out there, I could be flat on my back for the next 2 days*.” [male patient, 60–69 years old]
	22. “*When I went back to work the same kind of thing started to happen to me. And I didn't get along with one company that I worked for because the guy basically said, you're lazy. I'm like, I am not. Everything gets done. That's ridiculous. Because people don't understand what you're going through. And I don't expect another person to know what's happening to me, but it does happen. And it's visible to other people at times, they just don't understand it. And I would bartend, and people would say, oh, you need to smile more. I'm like, I can't*.” [male patient, 50–59 years old]
	23. “*I used to be very physically active outdoors and that is not a possibility right now, so I'd like to be able to get back to that. Not using a mobility aid would be nice. I just want to be able to do things that brought me great joy and I could do those again if my symptoms were better*.” [male patient, 50–59 years old]
Impact on caregivers	24. “*I work from home, but it really impacted the number of hours I could work because in between taking care of her and at various points feeding and bathing and even just having to get her to the bathroom. That really takes over your life and then I probably spent 100 h on the phone with the insurance company. So, it's been a lot*.” [female caregiver, 50–59 years old]
	25. “*Especially in those first few years, we didn't plan ahead because my plans were usually to help her. And we just did that for a long time. I work just part‐time now*.” [female caregiver, 60–69 years old]

#### 3.3.1 First Symptoms

Participants shared their experiences of having to cope with debilitating symptoms while simultaneously seeking a diagnosis and impactful treatments, often waiting years before getting diagnosed. It was described how symptoms, such as a drooping eyelid or eye‐crossing, may be first recognized by another person who recommends the patient sees a doctor. One caregiver recalled the rapid, noticeable onset of symptoms in the patient she cares for (Table 2, Quote 1).

Muscle tiredness and weakness during physical activity were highlighted by participants as debilitating. The fatigue experienced by many was described as insurmountable, making it difficult to get out of bed; it also interfered with daily tasks such as brushing teeth and combing hair. One participant recalled their feelings of exhaustion (Table 2, Quote 2).

Difficulty chewing and swallowing can lead to rapid weight loss and eating‐related anxiety in patients with gMG. The decision to limit or stop eating contributed to weight loss, among other issues, in some patients, and the potential to choke due to swallowing challenges was described as a “crisis” that prompted a search for a diagnosis. A participant recalled her initial problems swallowing, and how this impacted her weight (Table 2, Quote 3). Conversely, some participants said that they gained weight due to inactivity related to fatigue.

The symptom of facial weakness affects the ability of patients with gMG to breathe and speak clearly, but it can be mistaken for other illnesses, such as stroke, often prompting immediate medical attention. One participant recalled a doctor making that assumption on noticing this symptom (Table 2, Quote 4).

#### 3.3.2 Reaching a Diagnosis

Participants reported difficulties in receiving an appropriate diagnosis and described feeling overwhelmed, but relieved, when the diagnosis was finally made. Limited access to knowledgeable and specialized care leads patients with gMG to cycle through multiple healthcare professionals (HCPs), including primary care physicians and advanced practice providers (APPs) such as nurse practitioners and physician assistants. Referral to a specialist (neurologist or neuromuscular specialist) who recognizes the symptoms of gMG was often difficult to achieve (Table 2, Quote 5).

While seeking care from an APP was common, these HCPs are not always knowledgeable about disease presentation. The difficulty in accessing a specialist led some participants to experience delayed or incorrect diagnosis, and variable diagnostic journeys (Table 2, Quote 6).

Of the 13 PERC participants with gMG, only two received a correct diagnosis following their initial symptoms; the remaining 11 were misdiagnosed as having eye misalignment (*n* = 1 [6.3%]), astigmatism (*n* = 1 [6.3%]), hypertension (*n* = 1 [6.3%]), stroke (*n* = 2 [12.5%]), Bell's palsy (*n* = 1 [6.3%]), multiple sclerosis (*n* = 1 [6.3%]), anemia (*n* = 1 [6.3%]), asthma (*n* = 2 [12.5%]), or chronic obstructive pulmonary disease (COPD) (*n* = 1 [6.3%]). This delay to receiving a correct diagnosis due to limited access to knowledgeable and specialized care was particularly common among three of the four Black/African American patients, one of the two seronegative patients, and two of the patients aged ≥ 65 years. Those from rural populations said they often had to travel long distances to access care and effective treatments. Seronegative patients found it especially hard to receive a diagnosis when their laboratory results did not meet the definition of gMG. One participant recalled such an experience after seeing a primary care physician, ophthalmologist, and migraine specialist (Table 2, Quote 7).

This limited access to specialized care also meant that some patients required emergency care, where a correct diagnosis was also rare, and they were often treated for their symptoms rather than for gMG (Table 2, Quote 8).

#### 3.3.3 Treatment Experience

Barriers to positive treatment outcomes included failure of HCPs to initiate or escalate treatment as appropriate, difficulty articulating symptoms, issues related to health insurance and access to treatments, communication disconnect between patients and HCPs, negative experiences, fear of side effects, and type of gMG diagnosis. One participant described her battle with obtaining basic treatments (Table 2, Quote 9), and another participant described the side effects experienced by the patient she cares for (Table 2, Quote 10).

Participants said the immediate relief achieved with treatment made them feel initially optimistic; however, this optimism waned after first‐line therapies failed to produce a long‐lasting effect (Table 2, Quotes 11–12). However, one participant recalled the positive outcome of using a new treatment (Table 2, Quote 13).

A preference for at‐home and self‐administered infusions was expressed by participants, as going to hospital was viewed as a draining experience. The fear of treatment‐related infection was also something that prevented patients from pursuing other options (Table 2, Quote 14).

Participants mentioned that health insurance may prevent them from receiving treatment. One described her experience when she finally received treatment (Table 2, Quote 15). In some cases, health insurance issues prevented patients from receiving or continuing with newer, more advanced treatments such as biologics.

Therapeutic options for seronegative patients are often limited. Seronegative patients also reported that they are denied treatment options because their insurance does not cover certain medications. One participant explained the impact of being seronegative (Table 2, Quote 16).

Participants described situations where a disconnect in communication between patients and HCPs impacts confidence and adherence to a given treatment plan, and how difficulty in articulating symptoms and treatment goals serves as a barrier to treatment (Table 2, Quote 17).

It was also noted by both patients and caregivers that there is limited discussion with HCPs around treatment attributes, such that patients may not fully understand the different treatment options.

#### 3.3.4 Impact and Unmet Needs

Participants reported that they struggled to perform routine activities (e.g., household chores, spending time with family and friends, outdoor activities, reading) and that they required substantial assistance from caregivers for routine tasks. Most participants expressed dissatisfaction with the level of disease control because of fluctuating symptoms and waning efficacy of standard‐of‐care treatments; they felt this negatively impacted their quality of life. Setbacks, such as disease flares after a stable period, were considered discouraging. Patients struggled to regain disease stability, which was identified by the participants as the most important treatment goal overall (Table 2, Quote 18).

The need for more effective treatment options to reduce symptom severity and to better manage gMG is evident, with participants sharing their fears, concerns, and experiences with side effects in relation to standard‐of‐care treatments. Patients hoped treatment would help them to “return to normalcy” and activities they enjoy (e.g., running, spending time outdoors, spending time with family and friends in active settings), but had low expectations of treatments (Table 2, Quote 19).

Participants believed there is a trade‐off between quality of life and flexibility in treatment options and were prepared to modify their lives according to medication requirements. There was a strong desire among the patients for control of medication administration and to self‐administer at home, as opposed to relying on hospitals, as well as to decrease the number and frequency of medication administrations (Table 2, Quote 20).

Detrimental effects on physical health were mentioned, with participants reporting debilitating fluctuations and triggers in symptoms on a daily basis. Heat sensitivity prompted them to lose vision, become fatigued, or even faint, and exhaustion in the afternoon or evening sometimes exacerbated symptoms, such as physical weakness, inability to smile, and vision weakening or impairment. One participant recalled this effect in the summer (Table 2, Quote 21).

Participants also indicated mental health struggles, including stress and anxiety leading to social isolation, damage to close relationships, strained marriages, and depression (Table 2, Quote 22).

The transition from having good health to living with a chronic illness can be a *“traumatic”* experience for patients with gMG (see Supporting Information). Persistent symptoms, such as fatigue and weakness, impact patients' ability to enjoy events and maintain a social life. One participant described this decline (Table 2, Quote 23).

#### 3.3.5 Impact on Caregivers

The prework survey and focus groups indicated that caregivers were negatively impacted by the needs of patients with gMG, especially when symptoms are unmanaged. Discussions in the focus groups revealed that demands on caregivers included having to be available at all hours of the day to help with essential activities such as feeding and bathing and to serve as the voice of the patient. The constant and changing nature of gMG impacted the social and professional lives of caregivers, and spending extended time on the phone with insurance companies can be draining (Table 2, Quote 24). Another caregiver recalled the impact on both her work and her ability to plan (Table 2, Quote 25). Other burdens expressed by caregivers included difficulties socializing with friends and family and the need to limit heat exposure due to the negative impact on the patient that they are caring for.

## 4 Discussion

This study identified key insights around the impact of gMG on patients and caregivers, including their primary treatment goal of achieving stable disease, and the need to improve access to specialist care, achieve earlier diagnosis, prioritize patients’ preferences in disease management, and develop treatments that improve outcomes without additional burden.

The insights from the study led to recommendations that focus on the patients with gMG and ways in which education about the disease can help improve diagnosis, treatment access, and patient perception around their treatment choices. This includes providing patients with tools that inform them about treatment options and help them articulate goals and needs, so they can effectively engage in treatment decision‐making. One of the key findings driving the recommendations was the disconnect in communication between patients and their HCPs and the requirement for education to bridge this gap. There is a need for patients to better understand the course of their disease and the range of treatments available; they also require an improved understanding of how these treatments work (i.e., benefits and possible side effects), to abate their fears. For HCPs, educational needs were also identified around gMG presentation, earlier diagnosis, and prevention of misdiagnosis through recognition of first symptoms, along with an improved understanding that patients’ priorities can differ from theirs, and that patients often want to balance treatment options with effects on quality of life, as well as current and newer treatment options. The insights from this study also led to recommendations to ensure improved access to care for underserved populations and ensure research considers the burden of gMG on patients and incorporates relevant patient‐reported outcomes.

To date, only a few studies have examined the patient perspective of living with gMG. A recent targeted literature review highlighted that while the MG activities of daily living is an insightful tool for gMG symptomology, it does not directly measure other aspects of gMG [32] such as quality of life or general functioning. Another recent review/opinion paper by Gilhus et al., developed through informal consensus discussions between treating neurologists and patient representatives, highlighted the domains of disease that are important to patients, but are less important to treating neurologists [24]. In line with our findings, key topics and areas of unmet needs relate to daily function, symptoms, cognitive function, treatment availability, treatment burden, and misdiagnosis. In addition, that study identified challenges with managing comorbidities and issues around reproduction and parenting that were not addressed in the current study. Gilhus et al. revealed that patients want to better understand how the disease and subsequent treatments will affect daily life and generally seek further information and follow‐up; as such, the study concluded that there is a need for more patient‐centered studies to better understand their overall needs [24].

A patient‐led study by Law et al. gathered insights from patients and their caregivers across Europe and the United States [17]. Fourteen patient advocates living with gMG worked together to collect insights retrospectively from qualitative data obtained using three data sources: a patient council meeting, data reported in the literature, and a global qualitative research study of people affected by gMG. Several key areas identified are in line with our own findings and include the issue of fluctuating symptoms affecting a sense of disease stability, adapting to a life with limitations and trade‐offs with daily activities, suboptimal disease control and the burden/unpredictability of flare‐ups, a general disconnect with HCPs, and mental health issues [17]. A further US‐based study conducted concept‐elicitation interviews with 28 patients with gMG [18]. The study also identified that fluctuating and unpredictable symptoms have a major impact on patients' quality of life and that achieving symptom stability is an important patient goal. The authors emphasized the need to recognize the impact of symptom fluctuation and to consider it in treatment decisions [18].

The present study's findings concur with other studies' results and underscore the need for more research into how patients, caregivers, family, and friends perceive the experience of living with gMG, including identifying factors that are important to them. Future research should involve an evaluation of shared decision‐making and how well HCPs consider patient values when making treatment decisions. Given the importance placed by patients on fluctuating symptoms in our study and others [17, 18], a better understanding is needed of how patients define disease control versus how it is defined by HCPs. Another important area for research is what patients know and feel about available medications, and particularly how they perceive the convenience and burden of different treatments. Despite the small number of caregivers in our study, they provided valuable insight into living with gMG and should be included in future patient‐centric studies, as they often act as the patient voice and understand first‐hand the challenges faced by patients who have gMG.

A key strength of our study is that the participant insights were collected first‐hand and were subject only to our interpretation. In addition, each participant was able to contribute extensively through their answers to open‐ended questions, perhaps providing insights and anecdotes that might not otherwise be captured in closed‐ended survey questions. However, the small sample size may mean that the perspectives shared during this study and the findings described here may not be generalizable to the wider population of people living with gMG. The study population included only patients with a disease duration of either up to 10 years or more than 21 years; there were no patients with a disease duration of 11–20 years. Additionally, there was a risk of inaccuracy in self‐reporting on antibody status due to lack of knowledge.

## 5 Conclusions

Our findings confirm that numerous unmet needs exist for gMG patients, including improving access to specialist care, expediting diagnosis to offer earlier treatment, optimizing disease management (including understanding and prioritizing patient needs and preferences), and developing more effective treatments that reduce disease burden without increasing treatment burden. We also found that caregivers experience high demands on their time and considerable burden. Insights from the gMG PERC led to a list of recommendations identifying patient and HCP educational needs and guiding communication strategies, with the aim of improving the quality of life of those with gMG and their caregivers.

## Author Contributions

All authors take full responsibility for the entire manuscript content, integrity of the data, and the accuracy of the data analysis. Study concept and design: Gabrielle Geonnotti, Jacqueline Pesa, Wesley Peters, Melina Taylor, Zia Choudhry, Oluyemisi Falope. Data collection: Wesley Peters, Melina Taylor. Analysis and interpretation of data: Gabrielle Geonnotti, Jacqueline Pesa, Wesley Peters, Melina Taylor, Zia Choudhry, Oluyemisi Falope, Marquetta Price, Lucy Baxter, Bruce West, Lisa Shea. Original drafting of the manuscript: Jacqueline Pesa, Wesley Peters. Critical revision of the manuscript: Gabrielle Geonnotti, Jacqueline Pesa, Wesley Peters, Melina Taylor, Zia Choudhry, Oluyemisi Falope, Marquetta Price, Lucy Baxter, Bruce West, Lisa Shea. All authors read and approved the final manuscript.

## Ethics Statement

The study was conducted in accordance with the Helsinki Declaration of 1964 and its later amendments. This study was classified as market research, and institutional review board approval was not required.

## Conflicts of Interest

Jacqueline Pesa, Gabrielle Geonnotti, Zia Choudhry, Oluyemisi Falope, and Lisa Shea are employees of Janssen Pharmaceuticals US Inc. Titusville, NJ, USA. At the time of this study, Wesley Peters and Melina Taylor were employees of CorEvitas, which is now Evidera Inc. a part of Thermo Fisher Scientific Inc. Wilmington, NC, USA, which provided analytical services funded by Janssen Pharmaceuticals. Marquetta Price, Lucy Baxter, and Bruce West have no conflicts of interest to disclose.

## Supporting information

Supporting information.

## Data Availability

The data that support the findings of this study are available on request from the corresponding author. The data are not publicly available due to privacy or ethical restrictions.
