# Supplementary material for: Design of and Early Insights From a Generalized Myasthenia Gravis Patient Engagement Research Council
Source: Health Sci Rep. 2024 Dec 17;7(12):e70230. doi: 10.1002/hsr2.70230 (PMC11652384; doi:10.1002/hsr2.70230)
Supplement: Supplementary file 1 — Supporting information. [file HSR2-7-e70230-s001.docx]

**Supporting information**

**My journey with generalized myasthenia gravis: a patient experience narrative**

1988 was the year my life changed forever. I was 14 years old, in my best health. I took part in track, running was my passion. During the summer as I was preparing to go into eighth grade, I began to experience falls. My legs would give out and I would fall once or twice on each run. I thought to myself, “What’s going on, why do I keep falling?” I would get up, dust myself off and keep running. As the summer advanced I noticed my neck muscles felt strange, sore like I had slept on the wrong side of the bed and it was a constant struggle to keep my neck up. I also began to experience intense headaches daily. I was shy and frightened and felt isolated and depressed because I couldn’t understand what was happening to me. I also didn’t want to tell my parents; the battle was being fought alone.

Summer ended and I began eighth grade full of fear as my symptoms were getting worse and worse. By this time, I wasn’t able to eat meat because my neck was so weak that chewing became a struggle. Though my leg muscles were weak, I was still able to run. I joined the cross-country team, but began to lose my balance and kept falling. Instead of telling the coach or anyone in authority, I kept silent and quit the team.

October 28, 1988, was the day I knew I needed to tell someone, but I was so scared and thought no one could help me. After school I would always take the bus home, but that day my legs gave out and I fell from the top of the bus, hurting my head, shoulders and back. I walked home, limping in pain. My mother saw me coming down the street, and frightened, she asked me, “What’s wrong – did someone give you anything at school, you don’t look well?” I told her I was fine and started up the stairs but could barely lift my feet. With only three steps to go, my legs gave out and I fell down the stairs. My mother yelled in fear but I told her, “I just lost my balance, I'm okay.” She helped me inside the house where I went straight to my room. I didn’t know what to do. I was in dire need of help but I kept silent. In the desperate mindset I was in, I decided that I couldn’t go back to school and ended up making myself sick enough to have to miss the next day.

Saturday came and we had a family barbecue. My mother, by this time, was making sure I would eat because she had noticed I wasn’t eating. By this time, I couldn’t eat solid foods and my neck muscles became so weak that I was scared to eat. My mother sat next to me and said, “You need to eat this taco right now in front of me.” I told her I wasn’t hungry, but she insisted I had to eat it. I was in a panic; I knew I couldn’t swallow. I took a bite of the taco; my jaw was weak with no strength to chew. As I tried to swallow, the food got stuck in my throat. I began to choke. My breathing was labored; I couldn’t get air into my lungs. My parents immediately called an ambulance, and as we waited, I felt thick mucus start to engulf my nostrils and throat. Each breath was a struggle; I was so scared. I knew I had to fight to stay alive. Everything started to fall silent, my eyes became blurry, and my body felt like a million needles were being inserted all over. Everything went dark.

I opened my eyes four days later. I was in a children’s hospital, intubated; my hands were tied to the railing of the hospital bed as I had tried to pull the tube before I was sedated. When I woke up I was conscious and terrified. Being intubated and conscious was true torture. I felt like I was underwater, suffocating and trying to catch every breath, even though doctors are telling you to relax and let the ventilator breathe for you. I was admitted on October 29, 1988, and intubated until mid-November. As my body recovered, I was prescribed steroids and discharged with no specific diagnosis. I was so happy to finally come home, but when I tried to eat I started choking again, became unconscious, and awoke a second time to find myself intubated in the hospital. While in the hospital, I was able to eat. A few weeks later, I was discharged. I was told by the treating doctor that I was just experiencing a severe form of influenza. He recommended I eat in smaller bites and rest between meals. In my heart, I felt like this was something more than just a bug; something was really wrong. Unfortunately, my parents and I didn’t know how to be advocates – we didn’t ask questions; we did what we were told. I left the hospital feeling hopeless.

Instead of going straight home, we went to a restaurant near the hospital. Once again, when I started to eat, I began to suffocate. My body began to shake uncontrollably; the mucus started building up. I was conscious and kept telling myself to stay calm. The ambulance arrived, and though I could hear them, by this time I couldn't see anything. There was a lot of commotion at the hospital and I heard the doctor’s saying, “We’re losing her!” At that moment, a neurologist came into the room, read my file, and said, “I think this girl has myasthenia gravis.” I was given medication, and within thirty minutes, I was able to open my eyes and sit up. After an hour I was able to walk a few steps, and it was easier to breathe. My generalized myasthenia gravis (gMG) diagnosis was confirmed when I tested positive for the anti-acetylcholine receptor antibody. I was given medication and told I would be getting surgery as soon as possible to remove the thymus. My new life started in January 1989.

One of the greatest things that ever happened to me was when I got back home after the surgery and my parents sat with me and said, “We are here to love you and support you in everything you need help with, but we will NOT treat you like a sick person. You have chores and schoolwork to complete – we will not treat you any different.” I was angry with them at first, but the sense of responsibility carried me through high school, though some days were harder than others. I remember one day I started to vacuum, but my legs gave out and I fell down. I had to lie on the floor for more than 20 minutes until I regained my strength. It took me over an hour, but I finished. The spirit of never giving up became real over the decades. My first and sophomore years, I only went to school part time. I had tutors helping me finish my work because double vision made writing and reading difficult. The school adapted to my needs, for example, by letting me leave class early so I could walk to my locker before all the kids got out, and letting me rest in the nurses office if I was feeling weak. I received my high school diploma in 1993 and felt so proud of my accomplishment. I was going to live a “normal” life. After graduation, I enrolled in college. These were the most fun years of my life; by then, I had gotten into a rhythm and kept a repetitive routine, which helped me know when I needed to rest. I gave myself goals to accomplish and didn’t limit my thinking.

I have now lived with gMG for most of my life, and menopause was particularly challenging as my early symptoms re-emerged. As a woman with more life changes to come, I am in a place where it is all unknown again. I have so many questions – what will happen as I mature further; will I go back to baseline – but I don’t see organizations talking about this. It feels like going full circle and again being unsure of what comes next. But as an adult I can self-advocate – I know I can share my needs with my neurologist and reach out to others who can help; this gives me the internal strength to know I can handle this and am not alone. Being older, I am more prepared, but also aware of being about to face new challenges and a new chapter that is as yet unknown. I have a great support system that I can rely on, so I know I am more equipped to handle the next chapter. I feel hopeful as I now have the tools to deal with whatever comes.

I want to use my story to let doctors know that it is not just about medications and treatment – they need to see the *whole* person, including their emotions and fears. A doctor, just saying "I hear you" – those three words show you matter and that they see you. It shows that they are on your side and it really changes everything. This is something every neurologist can do for their patients.
